# Structural comparison of substrate-binding pockets of serine β-lactamases in classes A, C, and D

**DOI:** 10.1080/14756366.2024.2435365

**Published:** 2024-12-23

**Authors:** Hyeonmin Lee, Hyunjae Park, Kiwoong Kwak, Chae-eun Lee, Jiwon Yun, Donghyun Lee, Jung Hun Lee, Sang Hee Lee, Lin-Woo Kang

**Affiliations:** a Department of Biological Sciences, Konkuk University, Seoul, Republic of Korea; b National Leading Research Laboratory of Drug Resistance Proteomics, Department of Biological Sciences, Myongji University, Yongin, Republic of Korea

**Keywords:** serine β-lactamase (SBL), β-lactams, substrate spectrum, antibiotic resistance, substrate-binding pocket (SBP)

## Abstract

β-lactams have been the most successful antibiotics, but the rise of multi-drug resistant (MDR) bacteria threatens their effectiveness. Serine β-lactamases (SBLs), among the most common causes of resistance, are classified as A, C, and D, with numerous variants complicating structural and substrate spectrum comparisons. This study compares representative SBLs of these classes, focusing on the substrate-binding pocket (SBP). SBP is kidney bean-shaped on the indented surface, formed mainly by loops L1, L2, and L3, and an additional loop Lc in class C. β-lactams bind in a conserved orientation, with the β-lactam ring towards L2 and additional rings towards the space between L1 and L3. Structural comparison shows each class has distinct SBP structures, but subclasses share a conserved scaffold. The SBP structure, accommodating complimentary β-lactams, determines the substrate spectrum of SBLs. The systematic comparison of SBLs, including structural compatibility between β-lactams and SBPs, will help understand their substrate spectrum.

## Introduction

Antibiotics dramatically extended the human lifespan by more than 20 years since their first clinical usage in the early 1900s^1^. Antibiotics still play an important role in modern medicine and healthcare systems: the use of antibiotics is not just for the treatment of bacterial infections but also for preventive treatment after surgeries and wounds and even for immunocompromised patients^2^. The versatile usage of antibiotics pressured the selection of multidrug-resistant (MDR) bacteria, which are currently one of the biggest threats to modern healthcare systems in terms of increasing casualty and medical costs^3^. The six leading MDR bacteria of *Escherichia coli*, *Staphylococcus aureus, Klebsiella pneumoniae, Streptococcus pneumoniae, Acinetobacter baumannii*, and *Pseudomonas aeruginosa* were estimated to have contributed to 4.95 million deaths worldwide in 2019^4^.

The most prescribed antibiotics worldwide are β-lactam antibiotics, which account for about 65% of all antibiotics^5^. The β-lactam antibiotics can be classified into penicillins, cephalosporins, monobactams, and carbapenems, all of which share the β-lactam ring with a varied additional ring and side chains^6^. The core scaffold of β-lactams mimics the terminal di-peptide of D-Ala-D-Ala of peptidoglycan in the bacterial cell walls, which is involved in the crosslinking of glycan strands of N-acetylglucosamine and N-acetylmuramic acid by penicillin-binding proteins (PBPs)^7^. The β-lactams bind to the PBPs competitively with the D-Ala-D-Ala moiety and inhibit the cell wall synthesis.

Enzyme-mediated resistance in β-lactam is caused by β-lactamases^8^. β-lactamases are a well-known resistant mechanism against the β-lactams, especially in problematic Gram-negative bacteria^9^, and divided into serine β-lactamases (SBLs) and metallo-β-lactamase (MBLs) depending on the type of catalytic moiety^10^. In the Ambler classification, SBLs are divided into classes A, C, and D. In the Bush-Jacoby-Medeiros functional classification, classes A and D are group 2, class C is group 1, and class B is group 3^11^. The orthodox classification is mainly by protein sequence^12^. In the study, the residues of class A, C, and D SBLs are numbered based on the standard numbering scheme of class A β-lactamases (ABL), the structural alignment-based numbering of class C β-lactamases (SANC), and the class D β-lactamase (DBL) numbering system, respectively^13–15^. The SBLs use two steps of acylation and deacylation in the hydrolysis of the β-lactam ring. The serine residue performs the nucleophilic attack on the carbonyl carbon of the scissile β-lactam amide bond and the resulting acyl-enzyme intermediate is cleaved by a water molecule to release a hydrolysed β-lactam product of penicilloate^16^.

SBL members show a highly varied substrate specificity and spectrum, and a rapidly increasing number of variants further increases the diversity. Evolutionarily, SBLs originated from actinomycetes D-alanyl-D-alanine (DD) peptidase, in which classes A and D SBLs are sister taxa and class C SBLs diverged before A and D^17^. In each class, members are further classified into subclasses based on the substrate spectrum of narrow, broad, or carbapenem-hydrolysing activity (Table 1)^18^. Thus far, there has been a limited study to compare classes A, C, and D members altogether. We compared several tens of classes A, C, and D and subclasses SBLs in sequence and structure, and proposed structural insights on the structural characteristics of the SBPs.

**Table 1.** The classification of β-lactamases^18^.

Catalytic moiety	Class	Subclass
Non-metal (Ser)	A	Classical narrow-spectrum (PSE, CARB)
		Extended-spectrum β-lactamases (ESBLs)(TEM, SHV, CTX-M)
		Class A carbapenemases (GES, KPC, SME, IMI/NMC-A, SFC)
	C	AmpC
		Extended-spectrum AmpC (ESAC)(CMY, DHA, Fox, Mox, ACC)
	D	Narrow-spectrum (OXA)
		Extended-spectrum β-lactamases (ESBLs)(OXA)
		Carbapenem-hydrolyzing class D β-lactamases (CHDLs)(OXA)
Metal (Zinc)	B	Subclass B1
		Subclass B2
		Subclass B3

PSE: Pseudomonas specific enzymes; CARB: Active on carbenicillin, TEM: Temoneira; SHV: Sulfhydryl reagent variable; CTX-M: Active on cefotaxime; first isolated at Munich; GES: Guiana-extended spectrum; KPC: *K. pneumoniae* carbapenemase; SME: *Serratia marcescens* enzyme; IMI: Imipenem-hydrolyzing β-lactamase; NMC: Not metalloenzyme carbapenemase; SFC: *Serratia fonticola* resistant to carbapenem; CMY: Active on cephamycins; DHA: Discovered at Dhahran, Saudi Arabia; Fox: Active on cefoxitin; Mox: Active on moxalactam; ACC: Ambler class C; OXA: Active on oxacillin.

## Materials and methods

### Sequence alignment

The source of the amino acid sequences of SBLs in the study is the National Centre for Biotechnology Information (NCBI). The sequences were aligned using T-coffee^19^. The percent identity matrix (sequence identity) was obtained through “seq_reformat” by T-coffee. T-coffee’s alignment results were visualised through ESpript 3.0^20^ and then analysed.

The GIs of SBLs are as follows: PSE-4; 13399765, CTX-M-9; 381353189, CTX-M-14; 66361079, CTX-M-15; 478247355, TEM-1; 22219359, TEM-34; 24158844, TEM-76; 73535720, SHV-1; 208435576, SHV-2; 30749600, SHV-11; 1545826456, GES-5; 529481960, KPC-2; 146386660, SME-1; 13096333, NMC-A; 6137620, SFC-1; 388326945, EC-1; 700588110, CMY-2; 99031672, CMY-10; 99031695, Mox-1; 635576524, Fox-4; 1357894348, ACC1; 1766844994, OXA-1; 28373599, OXA-10; 11514736, OXA-14; 2171220535, OXA-45; 532137762, OXA-23; 529281200, and OXA-48; 1720273141.

### Structure

The source of all SBL structures is the RCSB Protein Data Bank (RCSB PDB). The structures were aligned using PyMOL^21^. In PyMOL, the same class SBLs were aligned using “Action: to molecule (*/CA)”, and the other class SBLs were aligned using “super mobile, target, [object = name]”. RMSD values were also obtained through this alignment process. The chemical structures of β-lactams were prepared using ChemDraw Professional, version 16.0^22^.

The PDB IDs are as follows: PSE-4; 1G68, CTX-M-9; 4DE3, CTX-M-14; 1YLT, CTX-M-15; 4HBU, TEM-1; 1M40, TEM-34; 1LI9, TEM-76; 1YT4, SHV-1; 2ZD8, SHV-2; 1N9B, SHV-11; 6NFD, GES-5; 4GNU, KPC-2; 2OV5, SME-1; 1DY6, NMC-A; 1BUE, SFC-1; 4EQI, EC-1; 4KG2, CMY-2; 1ZC2, CMY-10; 1ZKJ, Mox-1; 3W8K, Fox-4; 5ZA2, ACC1; 6K8X, OXA-1; 1M6K, OXA-10; 1FOF, OXA-14; 7L5R, OXA-45; 4GN2, OXA-23; 4K0X, OXA-48; 6P96, KPC-2_apo; 5UL8, CTX-M-9_benzylpenicillin; 3HUO, CTX-M-9_piperacillin; 3Q07, CTX-M-9_cefoxitin; 1YMX, CTX-M-9_cefotaxime; 3HLW, CTX-M-14_ampicillin; 7K2Y, CTX-M-14_temocillin; 6UNB, CTX-M-14_cefotaxime; 4PM9, CTX-M-14_ceftazidime; 5TWE, CTX-M-15_ampicillin; 7U4B, TEM-1_imipenem; 1JVJ, SHV-1_meropenem; 2ZD8, GES-5_imipenem; 4H8R, GES-5_meropenem; 8V9G, KPC-2_ceftazidime; 6Z24, KPC-2_cefotaxime; 5UJ3, SFC-1_meropenem; 4EUZ, EC-1_oxacillin; 4JXG, EC-1_cefoxitin; 4KEN, EC-1_cefotaxime; 4KG2, EC-1_ceftazidime; 1IEL, EC-1_cephalothin; 1KVM, EC-1_imipenem; 1LL5, CMY-185_ceftazidime; 8JB8, Fox-4_ceftazidime; 5CHM, Fox-4_cephalothin; 5CHJ, Fox-4_cefoxitin; 5CGX, Mox-1_aztreonam; 4WBG, ACC-1_cefotaxime; 6K9T, ACC-1_cefoxitin; 6KA5, OXA-1_doripenem; 3ISG, OXA-13_meropenem; 1H8Y, OXA-23_meropenem; 4JF4, OXA-23_imipenem; 6N6X, OXA-24_oxacillin; 4F94, OXA-48_cefotaxime; 6PQI, OXA-163_imipenem; 7KHZ, OXA-48_doripenem; 6P9C, OXA-48_meropenem; 7KHQ, OXA-48_ertapenem; 6P99, OXA-48_imipenem; 6P97, OXA-51_doripenem; 5L2F, OXA-58_meropenem; 7VVI, OXA-82_doripenem; 8SQ7, OXA-109_doripenem; 8SQ8, OXA-163_meropenem; 7KHY, OXA-239_cefotaxime; 5WI3, OXA-239_doripenem; 5WI7, and OXA-239_imipenem; 5WIB.

## Results

### Conserved scaffolds of classes A, C, and D SBLs

**Figure 1.**
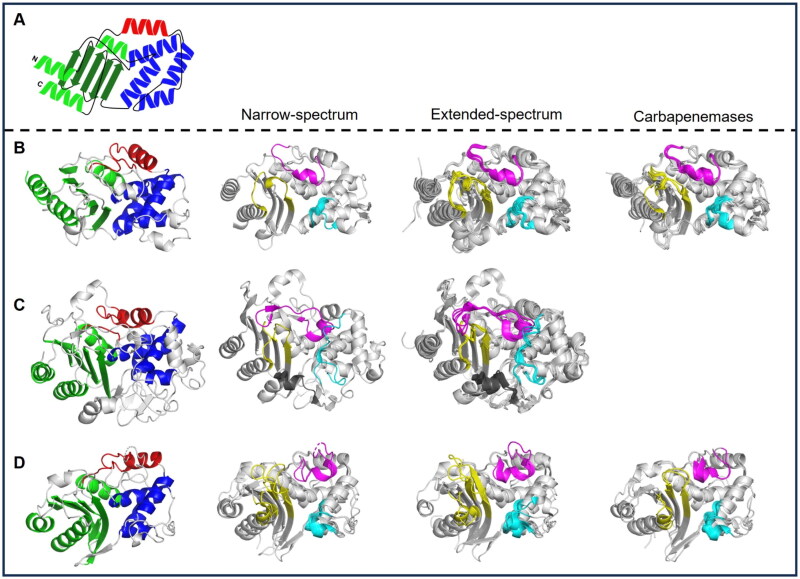
The Structural features of class A, C, and D SBLs. (A) The schematic diagram of the common structure and loops of SBLs. (B) The Structural features of class A SBLs. (C) The Structural features of class C SBLs. (D) The Structural features of class D SBLs. The common structures of SBLs for all classes of the first row. The three α-helixes on the left and one β-sheet are marked in green, the top one α-helix in red, and the four α-helixes on the right are marked in blue. The non-common parts between each class are marked with black lines. The superimposed structures of SBLs for each subclass of each class of the second to the fourth row. L1 is marked in cyan, L2 is marked in magenta, L3 is marked in yellow, and Lc is marked in grey. The structure of classical narrow-spectrum Class A (PSE-4; PDB ID: 1G68). The superimposed structures of extended-spectrum β-lactamases Class A (CTX-M-9; 4DE3, CTX-M-14; 1YLT, CTX-M-15; 4HBU, TEM-1; 1M40, TEM-34; 1LI9, TEM-76; 1YT4, SHV-1; 2ZD8, SHV-2; 1N9B, SHV-11; 6NFD). The superimposed structures of class A carbapenemases (GES-5; 4GNU, KPC-2; 2OV5, SME-1; 1DY6, NMC-A; 1BUE, SFC-1; 4EQI). The structure of AmpC (EC-1; 4KG2). The superimposed structures of extended-spectrum AmpC (CMY-2; 1ZC2, CMY-10; 1ZKJ, Mox-1; 3W8K, Fox-4; 5ZA2, ACC1; 6K8X). The superimposed structures of narrow-spectrum class D (OXA-1; 1M6K, OXA-10; 1FOF). The superimposed structures of extended-spectrum β-lactamases Class D (OXA-14; 7L5R, OXA-45; 4GN2). The superimposed structures of carbapenem-hydrolysing class D β-lactamases (OXA-23; 4K0X, OXA-48; 6P96).

Hundreds of SBL structures (apo structures, complex structures with hydrolysed products or inhibitors, variant structures, etc.) have been identified (Fig. S1 and Table S1)^23–247^. Among them, several tens of SBLs are selected for comparison, representing all three classes and the subsidiary subclasses in Table 1 ^35^^,^^55^^,^^59^^,^^62^^,^^73^^,^^74^^,^^77^^,^^96^^,^^111^^,^^119–123^^,^^132^^,^^151^^,^^163^^,^^172^^,^^175^^,^^179^^,^^203^^,^^221^^,^^238^. The amino acid sequences of the SBLs vary, and the sequence identity is as low as 9.5% among different classes (Fig. S2). Within the same class, A, C, and D SBLs have the average sequence identity of 43.8%, 53.8%, and 36.6%, respectively (Tables S2–S4) and show a conserved scaffold within each class (Figure 1).

**Figure 2.**
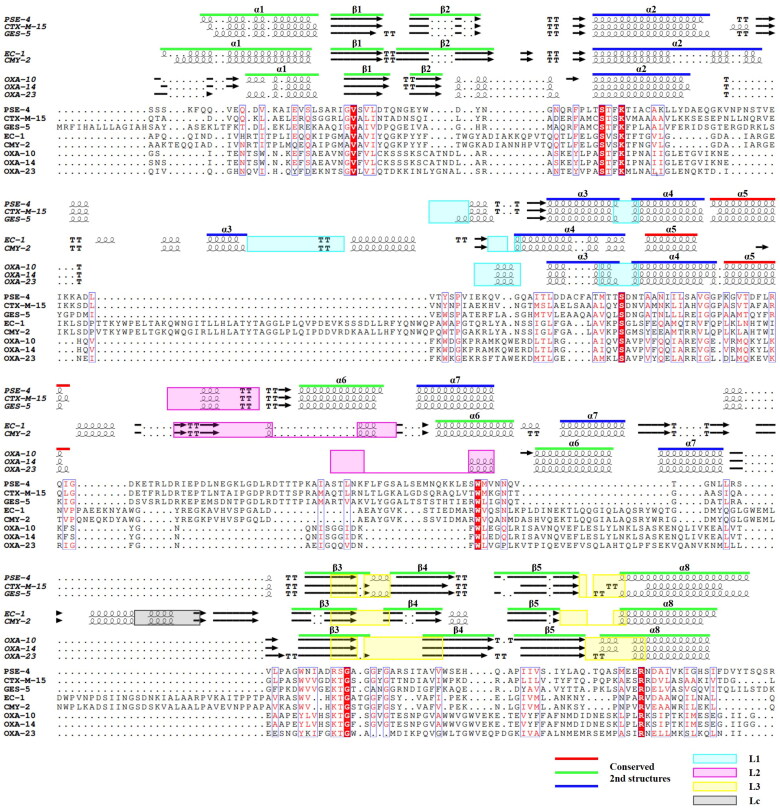
The structural sequence alignment of SBLs. The secondary structures of SBLs are shown at the top; the secondary structures are labelled based on the core scaffold of β-strands and α-helices. The common structure of the SBLs is marked in red, green, and blue lines above (α1–8, β1–5). The four loops L1, L2, L3, and Lc are marked as cyan, magenta, yellow, and grey boxes, respectively. Sequences of the following SBLs were aligned using T-coffee and ESPript 3.0.

We further compared the structures of SBLs among different classes. Although there is a low sequence conservation, we recognised the conserved secondary structures among the different classes (Figures 1 and 2). In the superimposed structures, the different classes of SBLs showed RMSD values from 2.68 to 5.44 Å (Table 2). Within the same class members, the RMSD values are as low as 0.68, 0.63, and 0.88 Å in classes A, C, and D, respectively. Even from the high RMSD values, different classes of SBLs share the core scaffold of eight α-helices (α1–8) and five β-strands (β1–5), which are shown in green, blue, and red, for easy recognition in Figures 1 and 2.

**Table 2.** The RMSD values of SBLs.

								RMSD (Å)
SBLs	PSE-4	CTX-M-15	GES-5	EC-1	CMY-2	OXA-1	OXA-14	OXA-23
PSE-4	0	0.73	0.95	4.00	4.12	4.32	4.04	4.02
CTX-M-15	0.73	0	0.75	5.44	5.04	2.68	2.77	3.43
GES-5	0.95	0.75	0	4.18	4.21	3.79	2.72	3.87
EC-1	4.00	5.44	4.18	0	0.45	4.86	4.99	3.25
CMY-2	4.12	5.04	4.21	0.45	0	4.44	4.96	4.04
OXA-1	4.32	2.68	3.79	4.86	4.44	0	1.12	1.18
OXA-14	4.04	2.77	2.72	4.99	4.96	1.12	0	1.03
OXA-23	4.02	3.43	3.87	3.25	4.04	1.18	1.03	0

Previously, the structures of SBLs have been considered as two domains of the α-helical domain, α/β domain, and a Ω-loop linking the two domains was recognised^140^^,^^248^. For the straightforward comparison of all three classes of SBLs, we divided the scaffold of SBLs into three domains: the α-helical domain, the α/β domain, and the Ω-loop domain. The α/β domain, including N-terminal and C-terminal parts, has mixed secondary structures of α-helices and anti-parallel β-strands, and the α-helical and Ω-loop domains mostly consist of α-helices. The substrate binding pocket (SBP) is formed from three loops, L1 (cyan), L2 (magenta), and L3 (yellow), from each domain, and only class C SBLs have an additional SBP-forming loop of Lc (grey) from the bottom between the α/β domain and α-helical domains (Figures 1 and 2). The L1, L2, L3, and Lc of SBLs are designated at structurally comparable positions from the superimposed structures. According to the naming in previous studies, the L1 exists in the α-helical domain, the L2 is a part of the Ω-loop in the Ω-loop domain, the L3 is in the α/β domain, and the Lc is in the hinge region of the additional domain in class C SBLs^172^^,^^249–252^. The R2 loop of class C SBLs is in the Lc, and the P-loop of Class D SBLs is in the L1 (Figure S3).

We further scrutinised the structure of each domain among classes. In the secondary structure of all SBLs, the α/β domain has a central β-sheet of antiparallel five strands, two α-helices and an α-helix on the other side of the β-sheet. The α-helical domain consists of a bundle of four α-helices. The Ω-loop domain connects α/β and α-helical domains via α-helices, including the SBP-forming flexible loop L2 (Figure 1). The conserved catalytic serine residue in the active site is located on the first helix of the α-helical domain. For each class of SBLs, there are differences in the number and size of secondary structures of each domain including the SBP-forming loops. The SBP-forming loops are directly related to the substrate specificity.

### Substrate-binding pockets

SBLs have a catalytic serine residue at the active site, and SBP is formed by the main three loops surrounding the catalytic residue. Structural comparison shows the characteristic structural features of SBP in each class. Three loops of L1, L2, and L3 form three sides of the SBP, with the fourth side open to the external space, except for class C SBLs having an additional Lc. All the three loops of classes A, C, and D members are different in size and conformation. In the structural comparison, class A SBLs have a short L1, a short L2, and a short L3; class C SBLs have a long L1, long L2, a short L3, and additional Lc; and class D SBLs have a short L1, a short L2, and a long L3 (Figure 1B–D).

### Class A SBLs

Class A SBLs can be classified into three subclasses, depending on the substrate spectrum: classical narrow-spectrum class A SBLs, extended-spectrum β-lactamases (ESBLs), and class A carbapenemases. The classical narrow-spectrum class A SBLs include PSE and CARB; ESBLs include TEM, SHV, and CTX-M; the class A carbapenemases include GES, KPC, SME, NMC-A, and SFC-1^18^^,^^253^^,^^254^. When the crystal structures of the representative class A members were superimposed^35^^,^^55^^,^^59^^,^^73^^,^^77^^,^^121–123^^,^^132^^,^^151^^,^^163^^,^^175^^,^^203^^,^^221^, the RMSD values among structures were between 0.12 and 1.12 Å (Table S5). The secondary structure elements of helices and β-strands are well conserved in all the class A SBLs, even in different subclasses.

**Figure 3.**
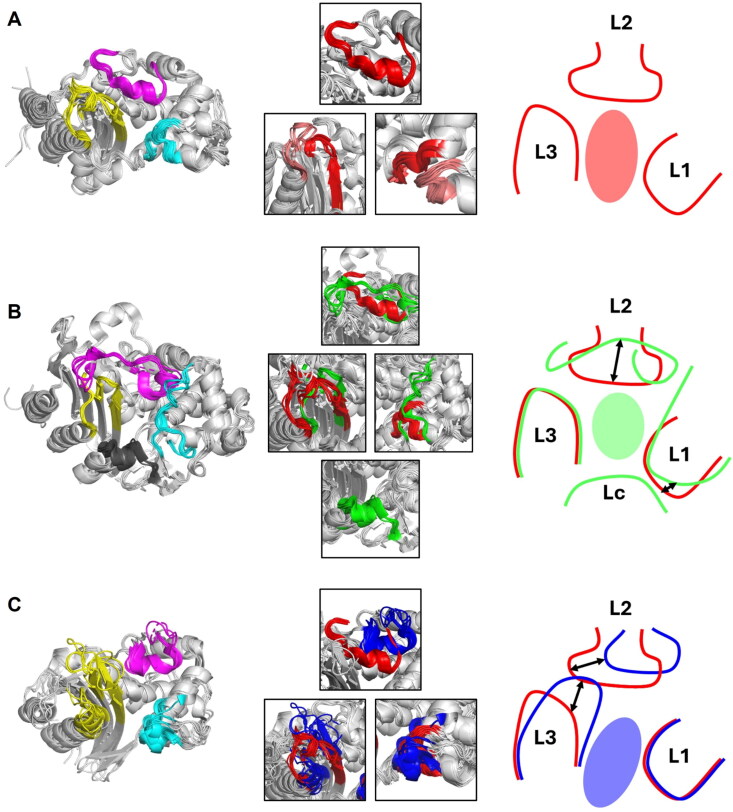
The comparison of the structures of L1, L2, and L3 of SBLs. The loops of class A, C, and D SBLs are marked in red, green, and blue, respectively. The differences in loop structures between classes A and C or D are indicated by two-way arrows. (A) The structures of loops of class A β-lactamases. The two loops constituting L1 are marked in red and pink, respectively. The two loops constituting L3 are marked in red and pink, respectively. (B) The comparison of the loops structures of class A β-lactamases and the loops structures of class C β-lactamases. (C) The comparison of the loops structures of class A β-lactamases and the loops structures of class D β-lactamases.

The class A SBLs have a well-conserved scaffold of nine α-helices and a β-sheet of five antiparallel β-strands (Figure 1B). According to the standard numbering of ABL for class A SBLs, L1 exists in residues 103–109 and 129–132, L2 in residues 163–176, and L3 in residues 234–242 and 267–272. With reference to the orientation shown in Figure 1(B), the three loops, L1 and L3 form the right-side and left-side walls, respectively and L2 forms the upper wall in SBP. The bottom side is open to solvent. The L1 exists between two inner helices of the α-helical domain, and the L3 is the loop between the innermost antiparallel strands in the α/β domain domain (Figure 3A). The L2 exists as a long loop between two helices in the Ω-loop domain. Although the conformation of three loops is well conserved in apo structures, the L2 and L3 loops showed conformational changes in the substrate-bound structures (Figure S4)^101^^,^^255^. In the ceftazidime-bound KPC-2 structure, L2 was opened due to the steric hindrance from the bound substrate, and L3 was moved inwards towards the bound substrate for stronger interactions.

### Class C SBLs

Class C SBLs are divided into two subclasses: AmpC and Extended-Spectrum AmpC (ESAC). ESAC includes CMY, DHA, Fox, Mox, and ACC^18^. In class C, amino acid sequences are more conserved than in classes A and D^256^; the average sequence identity is as high as 53.8%, which is 10.0% and 17.2% higher than that of classes A and D, respectively. When the crystal structures of the representative class C SBLs are superimposed^119^^,^^120^^,^^172^^,^^238^, the RMSD values are between 0.17 and 0.78 Å (Table S6). Like class A SBLs, the overall secondary structure scaffold of class C SBLs is well conserved.

The class C SBLs have the conserved scaffold of twelve α-helices and a central β-sheet of nine β-strands (Figure 1C). According to the standard numbering of SANC for class C SBLs, L1 exists in residues 112–125 and 149–152, L2 in residues 203–223, L3 in residues 315–323 and 341–346, and Lc in residues 287–296. The class C SBLs showed significant structural differences in the core scaffold, compared to class A and D SBLs; the four loops L1, L2, L3, and Lc form SBP instead of three loops. The additional Lc exists only in class C and forms the bottom wall of the SBP (Figure 3B). The L1 and L2 of class C SBLs are larger than those of class A and D SBLs.

With the additional domain having the Lc, class C SBLs are larger than class A and D SBLs. In the class C SBLs, the L1 is longer than that of class A SBLs and shifted upwards by the Lc at the bottom (Figure 1C). The L2 is also longer than that in class A SBLs. The long L2 shows a more complicated folding in a limited space between L1 and L3. Compared to the longer L1 and L2, the L3 has a similar size to that of class A SBLs. The Lc is formed with an additional long sequence of approximately 37 amino acids between the inner core helix in the α-helical domain and the end of the β-strand in the α/β domain, which forms the bottom domain to shift the α-helical domain upwards. The resulting SBP of class C SBLs is the smallest among SBLs.

### Class D SBLs

Class D SBLs (OXA) are divided into three subclasses; narrow-spectrum class D, Extended-Spectrum β-Lactamases (ESBLs), and Carbapenem-Hydrolysing class D β-Lactamases (CHDLs). Narrow-spectrum class D SBLs include OXA-1 and OXA-10; ESBLs include OXA-14 and OXA-45; and CHDLs include OXA-23 and OXA-48^18^^,^^257^. Class D SBLs have a high sequence identity of 73.1%–99.6% within the same subfamily, but that within different subfamilies is as low as 16.4%^258^. When the crystal structures of the class D members are superimposed^62^^,^^74^^,^^96^^,^^111^^,^^179^, the RMSD values among structures are between 0.16 and 1.18 Å (Table S7).

In the class D SBLs, the composition of secondary structures is well-conserved to have nine α-helices and a central β-sheet of seven β-strands (Figure 1D). According to the standard numbering of DBL for class D SBLs, L1 exists in residues 100–106 and 115–120, L2 in residues 151–167, and L3 in residues 216–231 and 255–263. The class D SBLs have the smallest overall size compared to the other class SBLs, but the three loops are different. The L1 has a similar size to that of class A SBLs but is more compact. The L2 also has a similar size to that of class A SBLs. However, the L3 is longer than that of class A SBLs (Figure 3C).

In the small and compact class D SBLs, the longer L3 in the α/β domain shifts the L2 to the right compared to that of class A SBLs (Figure 1D). The longer L3 showed more flexible conformations in both loop and β-strand parts, compared to that of other classes. In SBP, more space is available at the bottom (Figure 1D). Accordingly, substrates were bound more downwards in the open bottom space, compared to class A and C SBLs.

### Chemical structures of β-lactam antibiotics

**Figure 4.**
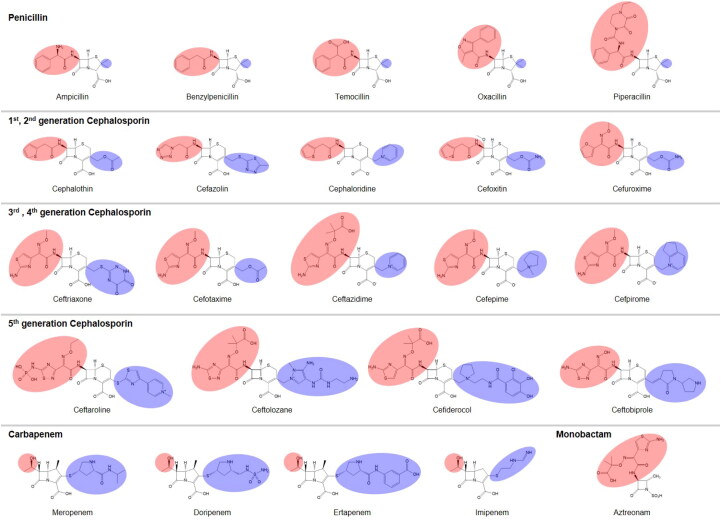
The chemical structures of the four major classes of β-lactams. The substituted R groups at the four-member β-lactam ring are shaded in red, and those at the five or six-membered dihydropyrrole ring or dihydrothiazine ring next to the β-lactam ring are shaded in blue.

**Figure 5.**
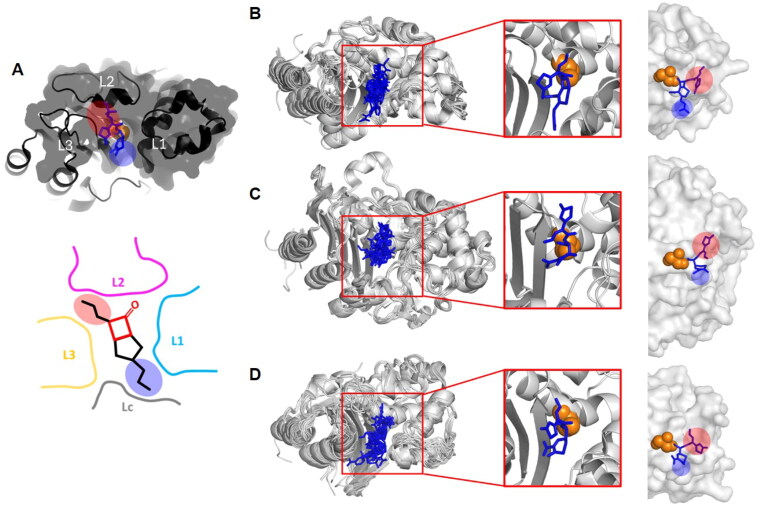
The structures of the substrate-binding pocket of SBLs. In the substrate binding pocket of SBLs, the R groups of the β-lactam ring part are shaded in red, and the R groups of the additional ring part are shaded in blue. The serine residue is marked with an orange sphere. (A) The common structure of substrate-binding pocket of SBLs. (B) The superimposed structures of class A β-lactamases in complex with substrates (CTX-M-9_benzylpenicillin; 3HUO, CTX-M-9_piperacillin; 3Q07, CTX-M-9_cefoxitin; 1YMX, CTX-M-9_cefotaxime; 3HLW, CTX-M-14_ampicillin; 7K2Y, CTX-M-14_temocillin; 6UNB, CTX-M-14_cefotaxime; 4PM9, CTX-M-14_ceftazidime; 5TWE, CTX-M-15_ampicillin; 7U4B, TEM-1_imipenem; 1JVJ, SHV-1_meropenem; 2ZD8, GES-5_imipenem; 4H8R, GES-5_meropenem; 8V9G, KPC-2_ceftazidime; 6Z24, KPC-2_cefotaxime; 5UJ3, SFC-1_meropenem; 4EUZ). (C) The superimposed structures of class C β-lactamases in complex with substrates (EC-1_oxacillin; 4JXG, EC-1_cefoxitin; 4KEN, EC-1_cefotaxime; 4KG2, EC-1_ceftazidime; 1IEL, EC-1_cephalothin; 1KVM, EC-1_imipenem; 1LL5, CMY-185_ceftazidime; 8JB8, Fox-4_ceftazidime; 5CHM, Fox-4_cephalothin; 5CHJ, Fox-4_cefoxitin; 5CGX, Mox-1_aztreonam; 4WBG, ACC-1_cefotaxime; 6K9T, ACC-1_cefoxitin; 6KA5). (D) The superimposed structures of class D β-lactamases in complex with substrates (OXA-1_doripenem; 3ISG, OXA-13_meropenem; 1H8Y, OXA-23_meropenem; 4JF4, OXA-23_imipenem; 6N6X, OXA-24_oxacillin; 4F94, OXA-48_cefotaxime; 6PQI, OXA-163_imipenem; 7KHZ, OXA-48_doripenem; 6P9C, OXA-48_meropenem; 7KHQ, OXA-48_ertapenem; 6P99, OXA-48_imipenem; 6P97, OXA-51_doripenem; 5L2F, OXA-58_meropenem; 7VVI, OXA-82_doripenem; 8SQ7, OXA-109_doripenem; 8SQ8, OXA-163_meropenem; 7KHY, OXA-239_cefotaxime; 5WI3, OXA-239_doripenem; 5WI7, OXA-239_imipenem; 5WIB).

Three classes of SBLs have different SBPs to bind various chemical structures of β-lactam antibiotics (Figure 4). In the superimposed structures of β-lactams-bound SBLs, the binding conformation of β-lactams was all identical (Figure 5); the β-lactam ring part binds to the upper side of the SBP towards L2, and the additional ring part binds to the open space in the opposite bottom of the pocket.

β-lactam antibiotics include penicillins, carbapenems, cephalosporins, and monobactams, which have various side chains of R groups in both the four-member β-lactam ring (red shade in the figure) and the five or six-membered additional ring (blue shade) except monobactams (Figure 4). We compared the R groups among the different classes of β-lactams. In the β-lactam ring part, penicillins have medium-sized R group modifications, carbapenems have small ones, the 1st and 2nd generation of cephalosporins have medium-sized ones, and the 3rd, 4th, and 5th generations of cephalosporins and monobactams have large ones. In the consecutively linked additional ring part, R modifications are small in penicillins, large in carbapenems and the 5th generation cephalosporins, and medium-sized in the 1st, 2nd, 3rd, and 4th generations of cephalosporins. There is no additional ring in monobactams.

### Conformations of bound β-lactams

The structures of β-lactam-bound SBLs are superimposed to study the substrate recognition in the varied SBPs (Figure 5B–D)^23^^,^^65^^,^^69^^,^^74^^,^^77^^,^^84^^,^^94^^,^^98^^,^^101^^,^^107^^,^^118^^,^^122^^,^^157^^,^^169^^,^^177^^,^^189^^,^^203^^,^^211^^,^^238^^,^^245^^,^^246^^,^^255^^,^^259–267^. The bound β-lactams show a conserved conformation in the SBPs in terms of binding orientation; the R groups in the β-lactam ring towards L2 and the other R groups at the additional ring towards the bottom between L1 and L3.

Class A and D SBLs have an open space on the bottom between L1 and L3, but class C SBLs have Lc on the bottom side. Relatively, in terms of SBPs, class C SBLs have the smallest SBP due to the blocking Lc domain, class A SBLs have the biggest SBP, and class D SBLs have a narrow and down-shifted SBP (Figure 5B–D). The L2 is shifted upward in class D compared to classes A and C, so the SBP has an elongated form from the top to the bottom. Thus far, various modifications have been introduced as different R groups and positions in the β-lactam core scaffold for better efficacy. The R modifications, especially bulky ones, play an essential role in the substrate specificity in different SBLs.

### Substrate preference of SBLs for β-lactam antibiotics

We compared the kinetic parameters of SBLs in each class and subsidiary subclass members having a narrow spectrum, an expended spectrum, and a carbapenemase activity (Table 3)^96^^,^^214^^,^^268–283^. Table 3 generally shows that class A members prefer penicillins, class C members prefer the 1st and 2nd generation of cephalosporins, and some class D members have carbapenemases activities and little activity for the 3rd and 4th generation of cephalosporins.

**Table 3.** The kinetic parameters of β-lactam antibiotics for SBLs^96^^,^^214^^,^^268–283^^,^^286–288^.

.	SBLs																
β-lactams	Class A							Class C					Class D				
	Narrow	Expended			Carbapenemases			Narrow	Expended				Narrow		Expended	Carbapenemases	
	PSE-4	CTX-M-15	TEM-1	SHV-1	GES-1	KPC-6	NMC-A	AmpC	CMY-9	Mox-1	Fox-3	ACC-2	OXA-1	OXA-10	OXA-14	OXA-23	OXA-48
Penicillin																	
Ampicillin	35	3.8	28.6	18		2.2			0.011	0.15	0.161		25		1.957	0.2	2.418
Piperacillin		1.8		16	0.009	0.6			0.0014	0.0101		0.55				0.15	0.18
Benzylpenicillin			17.1		0.25		9.3			3.9		0.816		6.7			
Oxacillin						1.3				0.0202				5.3	1.367		1.368
Temocillin					0.0000005												0.0066
Cephalosporin																	
Cephalothin			0.331	0.9	0.17	2.4	15.2	16	5.3	100.4	10	23.1	0.3	0.25	0.357		0.226
Cephaloridine			1.36		0.12			0.35	0.083						0.042		
Cefazolin		2.9															
Cefoxitin					0.0013	0.0019	0.0623	0.32	0.83	0.0497	5.56	ND		0.00038			0.00026
Cefuroxime		3				0.69		0.001									
Cefotaxime	0.009	3.7	0.00277	≤ 0.001	0.04	0.33	0.3	0.002	0.96	0.008	1.061	0.001	0.15	0.019	0.038		0.01
Ceftriaxone		2.5				0.75		0.0025							ND		
Ceftazidime		0.0012		≤ 0.00005	0.0275	0.0091	0.0522	0.0003	0.0032	ND	0.231	0.007			0.00067		ND
Cefepime		0.03		≤ 0.004	0.00083	0.048		>0.001	ND	ND	0.00332	0.024	0.2		0.86	0.018	0.0011
Cefpirome								>0.0001	0.0092			0.24					
Ceftolozane					0.0023												
Cefiderocol								ND									
Ceftobiprole		14	0.0029	0.23										7.9			
Carbapenem																	
Imipenem					0.005	0.44	11.3	0.01	ND	0.0031	0.0035	ND		> 0.023	ND	0.45	0.369
Meropenem					0.007	0.45	2.75							> 0.012	ND	0.04	0.0062
Ertapenem					0.0075	0.29											0.0013
Monobactam																	
Aztreonam		0.11			0.0012	0.34	5.6			ND		ND					

**Table:** 

> 10
0 < *k*_cat_/*K*_m_ < 10
0.01 < *k*_cat_/*K*_m_ < 0
0.0001 < *k*_cat_/*K*_m_ < 0.01
0.000001 < *k*_cat_/*K*_m_ < 0.0001

NH: hydrolysis not detectable; ND: not determinable. Unit of *k*_cat_/*K*_m_ is μM^−1^s^−1^.

When we consider the class A SBLs as a reference to have a standard SBP, they have a high activity for penicillins. The class C SBLs have the smallest SBP and prefer the 1st and 2nd generation of cephalosporins of small-size substrates. The SBP of class D SBLs is downshifted, which has a limited space towards L2 and open space at the bottom. The class D SBLs show lower activity for the 3rd and 4th generation of cephalosporins with big R chains in the β-lactam ring and higher activity for the carbapenems with big R chains in the additional ring.

In the structural comparison of subclass members, there are few structural differences found in the secondary structures of helices and β-strands in numbers and sizes. Accordingly, different substrate specificity and spectrum of subclass members could be caused by subtle differences, such as more flexible conformations of the SBP-forming loops or substituted residues in SBP by point mutations. Previous studies showed the extended-spectrum TEM-10 was produced as a result of Arg164 to Ser point mutation from the narrow-spectrum TEM-1^284^, and the extended-spectrum OXA-11 as a result of Gly157 to Asp mutation from the narrow-spectrum OXA-10^285^.

## Discussion

SBL members of different classes have the distinguished structural characteristics of SBPs, which are essential to determine substrate preference for various β-lactam antibiotics. We compared three classes A, C, and D, and subclasses of SBLs in the primary sequence, the overall scaffold, the SBP-forming loops, and the bound conformations of substrate β-lactams in SBP.

Although classes A, C, and D SBLs have different SBPs, β-lactam antibiotics are bound to the SBPs in the same orientation, in which the side R chains of β-lactam rings bind in a pocket close to L2, and those in the additional ring to a pocket between L1 and L3. The two rings in the core scaffold of β-lactams are connected at a bent angle. The directly bound two rings in a tilted angle make them fit into the SBP with only one conserved direction (Figure 5).

In the β-lactam-bound SBL structures, both R side chains in the two consecutive core rings are bound outwards from SBP. In the conformation, diverse R chains could bind SBP with more flexibility by exposing themselves to solvent. The solvent-accessible positions of β-lactam antibiotics could be more easily modified to escape antibiotic resistance without losing binding affinity. On the contrary, the other side of the carbonyl group and the carboxyl group of β-lactams faces into the catalytic serine residue in the active site, which cannot be modified to mimic the canonical substrate D-Ala-D-Ala moiety of peptidoglycans.

One of the reasons that β-lactams have been one of the most successful antibiotics is that the target is peptidoglycan layers. The peptidoglycan layers exist either as the most outer layer of Gram-positive bacteria, or in the periplasm of the Gram-negative bacteria. The β-lactams have the advantage over some other classes of antibiotics in that they do not need to enter the cytoplasm, thus minimising the effects of the penetration efficiency of β-lactams to access their site of action. At the same time, it maximises the probability that the structural compatibility study between the chemical structures of β-lactams *in vitro* and SBPs represents the drug efficacy in vivo. The efficient penetration of β-lactams to the drug site of action makes the structure activity relationship study of β-lactams and β-lactamases a good example to study the drug resistance caused by the β-lactams hydrolases.

We studied the structural characteristics of class A, C, and D SBLs, especially SBPs. The systematic structural study elucidates the conserveness and differences among the three classes of SBLs. Despite the structural differences of SBPs, all SBLs recognise β-lactams in a conservative way. Different SBPs in classes A, C, and D SBLs show preferences for structurally compatible β-lactams. However, SBPs of the same subclass SBLs are well conserved in the secondary structures of helices and β-strands. More detail and specific structural study are necessary. The systematic structural analysis of all classes of SBLs will help us to understand the substrate specificity and spectrum of SBLs better.

## Supplementary Material

Supplementary_Material.docx

## Data Availability

The authors confirm that the data supporting the findings of this study are available within the article [and/or] its supplementary materials.
