# Supplementary material for: Structural comparison of substrate-binding pockets of serine β-lactamases in classes A, C, and D
Source: J Enzyme Inhib Med Chem. 2024 Dec 23;40(1):2435365. doi: 10.1080/14756366.2024.2435365 (PMC11703393; doi:10.1080/14756366.2024.2435365)
Supplement: Supplementary_Material.docx [file IENZ_A_2435365_SM2518.docx]

**SUPPLEMENTARY INFORMATION**

**Structural comparison on the substrate-binding pocket of serine β-lactamases in class A, C, and D**

Hyeonmin Lee^a¶^, Hyunjae Park^a^, Kiwoong Kwak^a^, Chae-eun Lee^a^, Jiwon Yun^a^, Donghyun Lee^a^, Jung Hun Lee^b^, Sang Hee Lee^b^*, and Lin-Woo Kang^a^*

*^a^Department of Biological Sciences, Konkuk University, 120 Neungdong-ro, Gwangjin-gu, Seoul 05029, Republic of Korea; ^b^National Leading Research Laboratory of Drug Resistance Proteomics, Department of Biological Sciences, Myongji University, 116 Myongjiro, Yongin, Gyeonggido 17058, Republic of Korea*

**Contents**

Table S1. The PDB IDs of class A, C, and D SBLs S3

Table S2. The sequence identity of class A β-lactamases S4

Table S3. The sequence identity of class C β-lactamases S5

Table S4. The sequence identity of class D β-lactamases S6

Table S5. The RMSD values of class A β-lactamases S7

Table S6. The RMSD values of class C β-lactamases S8

Table S7. The RMSD values of class D β-lactamases S9

Figure S1. The superimposed structures of class A, C, and D SBLs S10

Figure S2. The structural sequence alignment of SBLs S11

Figure S3. The position of the loops of class A, C, and D SBLs S12

Figure S4. The superimposed structures that apo structure (orange) and ceftazidime bound structure (purple) of KPC-2 S13

**Table S1.** The PDB IDs of class A, C, and D SBLs.

**Table S2.** The sequence identity of class A β-lactamases.

**Table S3.** The sequence identity of class C β-lactamases.

**Table S4.** The sequence identity of class D β-lactamases.

**Table S5.** The RMSD values of class A β-lactamases.

**Table S6.** The RMSD values of class C β-lactamases.

**Table S7.** The RMSD values of class D β-lactamases.

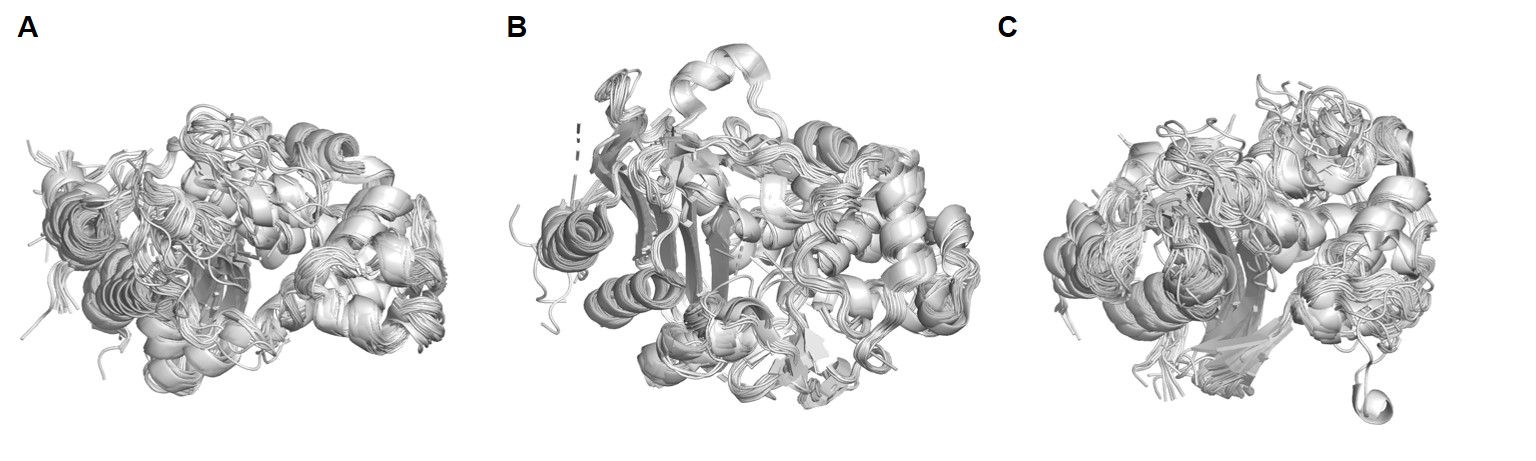


**Figure S1.** The superimposed structures of class A, C, and D SBLs. (A) The superimposed structures of class A β-lactamases. (B) The superimposed structures of class C β-lactamases. (C) The superimposed structures of class A β-lactamases. The PDB IDs are shown in Table S1.


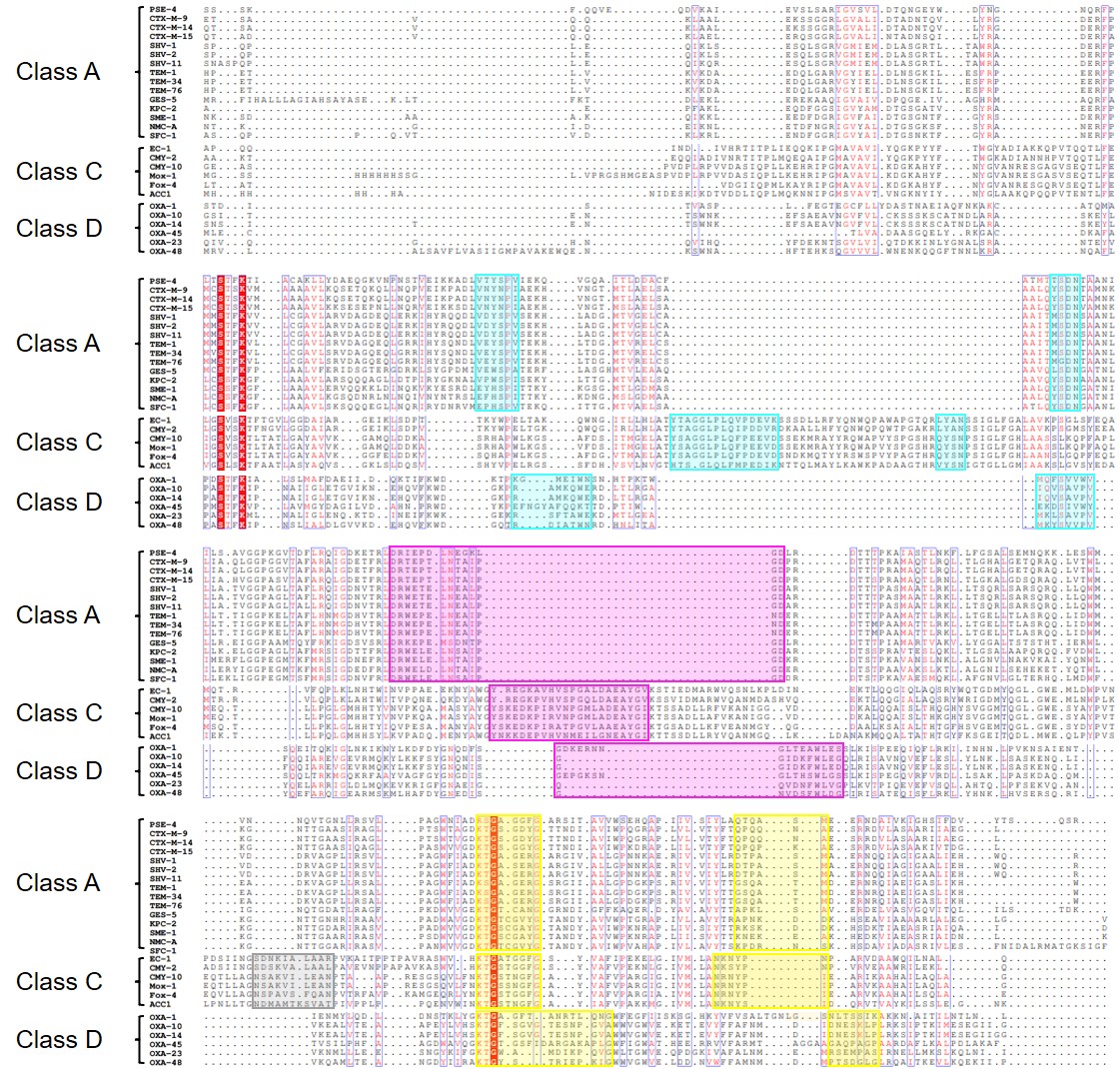


**Figure S2.** The structural sequence alignment of SBLs. Residues identical among all the amino acid sequences are marked in red. The four loops L1, L2, L3, and Lc are marked as cyan, magenta, yellow, and gray boxes, respectively. Sequences of the following SBLs were aligned using T-coffee and ESPript 3.0.


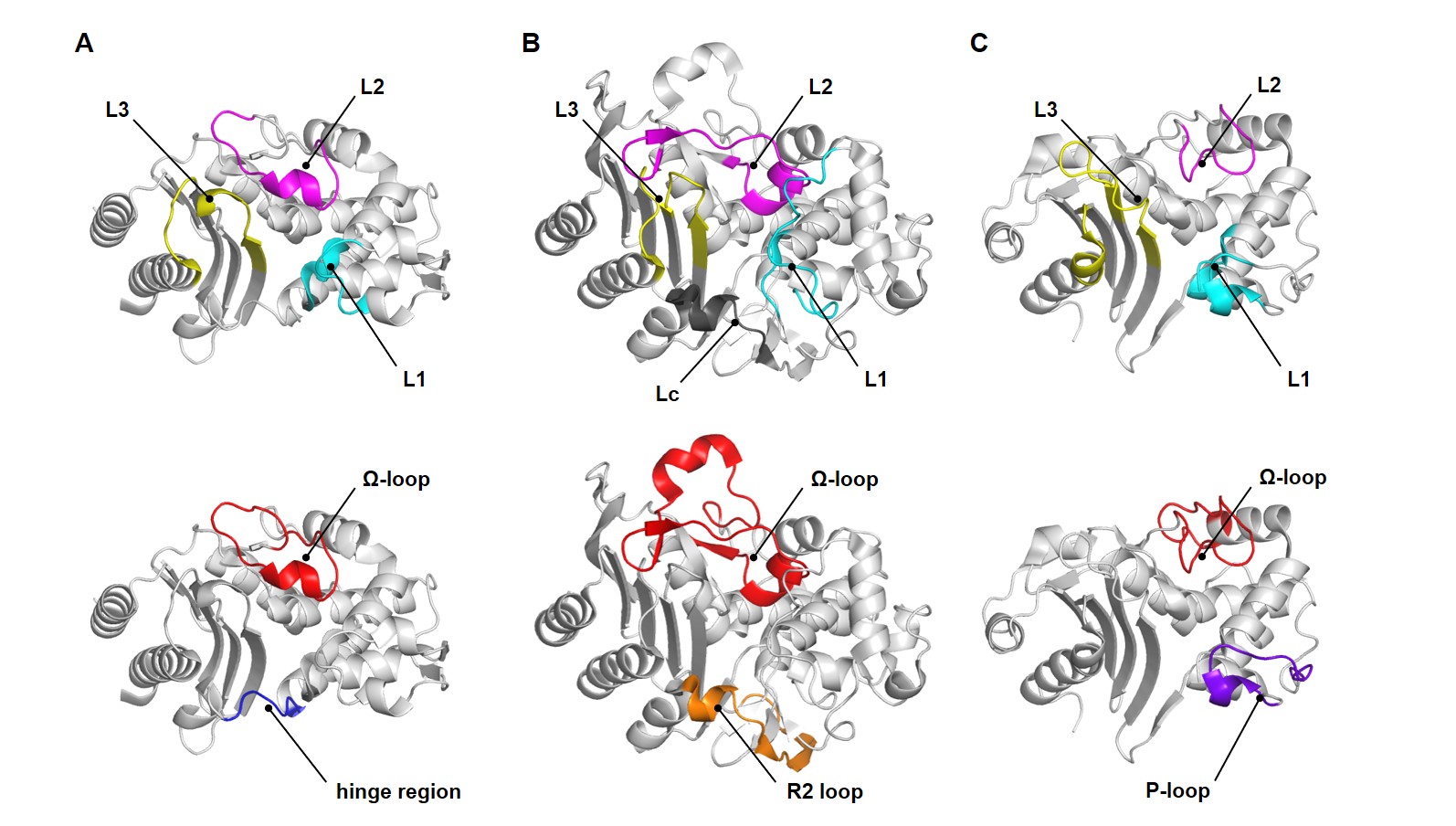


**Figure S3.** The position of the loops of class A, C, and D SBLs. L1, L2, L3, and Ω-loop are commonly marked in cyan, magenta, yellow, and red, respectively. (A) The position of the L1, L2, L3, Ω-loop, and hinge region (blue) of class A SBLs. (B) The position of the L1, L2, L3, Lc (grey), Ω-loop, and R2 loop (orange) of class C SBLs. (C) The position of the L1, L2, L3, Ω-loop, and P-loop (purple) of class D SBLs.


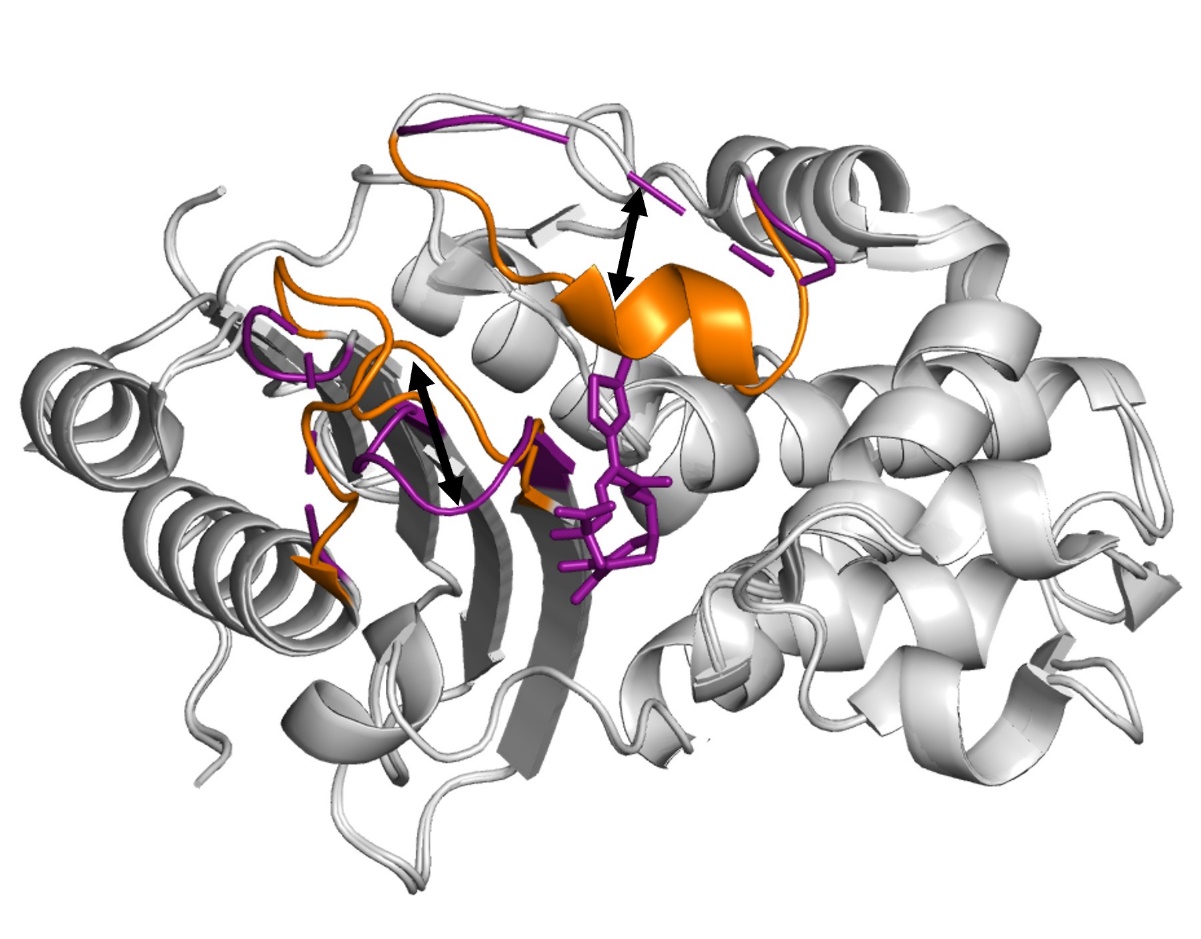


**Figure S4.** The superimposed structures that apo structure (orange) and ceftazidime bound structure (purple) of KPC-2 (KPC-2_apo; PDB ID: 5UL8, KPC-2_ceftazidime; 6Z24). The differences in loop structures are indicated by two-way arrows.
